# Patient safety education in undergraduate medical education through a global lens: a scoping review

**DOI:** 10.1186/s12909-025-07159-x

**Published:** 2025-04-16

**Authors:** Njoud Aldardeir, Qabirul Karan Abdullah, Linda Jones

**Affiliations:** 1 Centre for Medical Education, School of Medicine, University of Dundee, Dundee, UK; 2 Medical Education Department, Fakeeh College for Medical Sciences, Jeddah, 21461 Saudi Arabia; 3 Honorary senior lecturer, Kamuzu University of Health Sciences (KUHeS), Blantyre, Malawi

**Keywords:** Patient safety, Undergraduate medical education, Medical students, Medical teaching, Medical curriculum integration, Global differences in patient safety education

## Abstract

**Background:**

Patient safety, an organizing framework to minimize risks and harm to patients in healthcare delivery, is broadly accepted as a crucial component of global undergraduate curricula. The incorporation of Patient Safety Education (PSE) into medical curricula, as suggested by the World Health Organization (WHO) can be challenging and has been partially and inconsistently applied. Factors such as densely packed curricula, gaps in the evidence-base, under-prepared faculty, and low levels of organizational support have influenced implementation. This review highlights teaching and learning evidence relevant for such integration of PSE into undergraduate medical education and considers variations in educational advancement across different regions referred to as WEIRD (Western, Educated, Industrialized, Rich and Democratic) and Non-WEIRD countries.

**Methods:**

We followed the JBI protocol for undertaking scoping reviews to identify evidence-based gaps and recommend further research supporting integration of PSE into undergraduate curricula. Using PubMed, Scopus, ERIC, CINAHL and Cochrane library, 720 papers, from 2013 to 2023, were identified. Screening of titles and abstracts of 61 studies of PSE in undergraduate medical programs, 28 articles met the inclusion criteria. Descriptive statistical and thematic analysis for data extraction about curriculum design, learning and teaching interventions was conducted.

**Results:**

Findings showed 39% of 28 papers reviewed originated in European region, and 36% from the Americas. Over half (57%) of the selected studies used quantitative methods of analysis, 37.4% were mixed methods, and only 3.5% used qualitative approaches. A variety of methods were used including interactive (21.4%), experiential (14.3%) and technology-enhanced (17.8%) pedagogic strategies. The WHO curriculum guides, and the Institute for Healthcare Improvement (IHI) were the common sources shaping the content of the interventions. Four themes were identified, cultural and contextual considerations; curriculum structure/session design; student engagement/ application; leadership support and faculty training.

**Conclusions:**

Most publications and discourses emerged from WEIRD countries. Whilst outlining a range of pedagogical methods and curricular design, few explicitly referenced educational theories or addressed faculty development needs. Greater attention to cultural perspectives, local adaptation, efficacy of implementation strategies is needed globally. Research into longitudinal studies and impact on educational institutions will aid our understanding of how to promote, create and evaluate PSE across diverse countries.

**Clinical trial number:**

Not applicable.

**Supplementary Information:**

The online version contains supplementary material available at 10.1186/s12909-025-07159-x.

## Background

Patient Safety (PS) is in every ounce of medicine. PS, an organizing framework to minimize risks and harm to patients [1], has become a key discourse in medicine especially since the *Institute of Medicine* (IOM) report *“To err is human*” identified the worrying extent of medical errors in healthcare practice [2]. Unsafe healthcare causes more than 3 million deaths each year with up to 4 from 100 deaths in low-to middle-income nations caused by subpar care [3]. High income countries also encounter medical errors. For example, between 2018 and 2020, medication errors reported in Australia and USA were 13.1% and 12.6%, respectively [4].

Since 1998, the USA-based IOM recognised that improving safety requires collaborative effort and setting performance standards for educational improvement [2]. Recently in 2024, the WHO’s “*Global Patient Safety Report”* provided a comprehensive overview of seven strategic objectives for PS initiatives and advancements worldwide, underpinning the 2021–2030 Action Plan [5]. The fifth objective, focused on educating healthcare workers, and has received a score of 42 out of 100 in terms of global performance, indicating low levels of achievement [5].

Several key bodies and organisations responded to initial calls in the late 90s, by structuring and integrating Patient Safety Education (PSE) within curricula. Table 1 presents some of these well-known, seminal examples. It is noticeable that they originate from organizations in Western countries highlighting the paucity of contributions to discourses from the Eastern diaspora [6]. In this review, we refer to both entities using the more nuanced and respectful acronyms WEIRD (Western, Educated, Industrialized, Rich and Democratic) and non-WEIRD countries [7]. Additionally, although many of the papers considered report on wider healthcare education, we have assumed that medical education, the focus of this review, would be one component of their phenomena of interest.

**Table 1:** Examples of bodies or organizations who helped structure PSE

Organisations/bodies	Origin	Document
World Health Organization (WHO)	Global	Patient Safety Curriculum Guide: Multi-Professional Edition. WHO, 2011
Institute for Healthcare Improvement (IHI)	USA	IHI Open School curriculum (founded 1991)
General Medical Council (GMC).	UK	Outcomes for Graduates, 2018
Association of American Medical Colleges (AAMC)	USA	Core Entrustable Professional Activities (EPA) for entering Residency: Summary of the 10-School Pilot, 2014–2021
Accreditation Council for Graduate Medical Education (ACGME). Common Program Requirements. ACGME	USA	Common Program Requirements. ACGME, 2020
World Federation for Medical Education (WFME)	Global	Global Standards for Quality Improvement in Medical Education: The 2021 Revision. WFME, 2021
Royal College of Physicians and Surgeons of Canada	Canada	CanMEDS 2015 OTR Special Addendum, updated December 2016
German Association for Medical Education (GMA)	Germany	The Learning Objective Catalogue for Patient Safety in Undergraduate Medical Education, 2016

In 2010, Leape et al. identified medical educational reforms as one of the transforming concepts to healthcare improvement. They called to shift education focus from learning clinical and scientific information into developing knowledge, skills and behaviours for preparing safe practitioners [8]. A survey of north American medical schools reported rates of incorporating formal patient safety curricula in undergraduate programs as having increased from 12% in 2006 to 45.6% in 2012, still below half [9]. WHO 2024 statistics reported only one fifth of countries having incorporated PSE into undergraduate and postgraduate training with only 14% of these countries having integrated core PS abilities into their licensing and relicensing criteria [5]. This highlights a dearth of PSE worldwide, especially in Africa. Although other regions have initiated progress, it is surprising that none has yet reached an advanced level of PSE implementation [5].

The breadth of PSE knowledge and expertise held by medical schools is not comparable to that of other traditional medical subjects which have been taught for decades [10]. Although the “WHO Patient Safety Curriculum Guide” [11, 12] has mostly informed the structure, content, and delivery of PSE, it is still inadequately or inconsistently applied in medical schools globally [5, 13]. For example, in Brazil, a documentary analysis of medical curricula showed that PS is taught in a “fragmented manner” and none of the WHO topic themes were completely delivered. Many educational gaps remained manifest such as no inter or multidisciplinary guidance being established [14].

Discourses relating to PS have become more evident and diverse since the 1990s having evolved from identifying issues and significance of medical errors, to standards setting and structuring PS curricula and implementing integrated and innovative teaching approaches. Integrating and teaching patient safety in undergraduate medical programs, however, can be challenging due to densely packed curricula; discipline-based approaches; lack of leadership support and educator preparedness; resistance to change; gaps in best practice evidence [15]; and limited familiarity of PS requirements [16].

The current literature landscape suggests there are more studies on PS teaching in post-graduate medical education than undergraduate [17, 18]. The WHO global PS report emphasized interprofessional education to promote collaborative learning across different disciplines [5]. Additionally, the WHO curriculum guide recommends integration of PSE into each of healthcare disciplines curricula including medicine, nursing, etc [12]. This scoping review has selected integrating PSE into Undergraduate Medical Education (UGME) which addresses only one gap and recommendation. Insights gained from exploring innovative approaches to integrating safety principles into medical curricula may inform further research and adaptation to suit other health-professional contexts.

This scoping review was the first step in a larger PhD study aiming to enable and enhance recent focus on PSE in the Eastern Mediterranean region in general, and Kingdom of Saudi Arabia (KSA) in particular. Prior to delving into exploring different contexts and cultures, it felt important to establish an overview of current PSE teaching practices in medical schools globally by robustly examining how PSE is advancing and shaping both WEIRD and non-WEIRD countries. A 2024 paper identified the role of scoping reviews as helping to determine the extent of available evidence on specific issues; prioritize questions; identify contextual information; recommend actions, and explore implementation strategies through evidence surveillance [19].

### Aim of the study

This scoping review aimed to provide valuable insights informing design, implementation, and evaluation of PSE curricula offering well-rounded, evidence-based perspectives, considering diverse practices and evolving trends. We.

sought to identify gaps in the literature about undergraduate PSE and map current evidence-based practices for undergraduate medical teaching of PS globally. It will contribute to medical education research by providing a landscape of current gaps in PSE and identifying areas for future research. From practical perspectives, it can assist medical educators to design, integrate, and deliver PSE especially at the undergraduate level, by exploring a range of currently utilized teaching practices.

### Review question

The JBI protocol requires a research question(s) at the outset, ours was “How does the existing literature portray patient safety education in undergraduate medical curricula?” We added secondary questions:

- “What are the innovative teaching approaches used in patient safety education worldwide?”
- “How and when is patient safety taught in undergraduate medical curricula in WEIRD and non-WEIRD countries?”
- “How might the existing evidence base be utilized by non-WEIRD countries to promote PSE?”

### Eligibility criteria

The JBI protocol emphasises using the PCC (Population, Concept, and Context) framework to develop clear review questions and inclusion criteria [20].

#### Population

We considered population as undergraduate medical programs, whether or not preceded by a Bachelor of Science, as in North America or not, as in UK and the Middle-East. Other health professions, postgraduate programs and fellowships, or interprofessional PSE were excluded as beyond the scope of this study.

#### Concept

We searched for concepts related to curricular approaches incorporating educational interventions.

#### Context

PSE within university undergraduate settings worldwide.

Although some educational interventions and curricular modifications had been published prior 2013, our review chose to focus on the decade between 2013 to December 2023 as most likely to identify the latest innovative teaching approaches since the upsurge in PSE. We included only materials published in or translated into English. Papers that included evaluations of actual teaching interventions were included but studies exploring students’ perception and attitudes toward PS unrelated to curriculum design or teaching effort were not (Table 2).

**Table 2:** Inclusion and exclusion criteria

Criterion	Inclusion	Exclusion
Study design	Empirical or pilot studies including quantitative, qualitative, or mixed methods.	Reviews/ commentaries/ guides
Population	Undergraduate medical programs	Postgraduate, other health professions program, interprofessional
Type and scope of study	Educational activities to teach patient safety	Investigate students’ perception/attitude/ impact of PS education
Context	Educational (university) worldwide	Practical (hospital)
Language	English or translated to English	Other languages
Publication dates	2013–2023	< 2013

### Type of sources

We remained open to quantitative, qualitative, and mixed methods sources identified by JBI. Quantitative studies included randomized/non-randomized controlled trials, before and after, and interrupted time-series studies. Furthermore, observational studies including prospective and retrospective cohort and case-control studies were considered. Not only empirical research was included, small-scale pilot studies; often highlight pedagogic innovations, and grey literature can illuminate the nature of discourses and practices.

### Protocol

The JBI protocol [21] seeks to synthesize; map; identify existing evidence; capture key concepts and definitions; highlight gaps in knowledge; and as a pre-step to conducting a systematic review [22]. No protocol was registered with PROSPERO prior to conducting this review.

## Methods

This review was conducted in accordance with the JBI methodology for scoping reviews [21] aligning with the Preferred Reporting Items for Systematic Reviews and Meta-Analyses extension for Scoping Reviews (PRISMA-ScR) checklist [23] (Additional File 1).

### Search strategy

An initial search, using University of Dundee library search engine, was undertaken to identify articles on the topic and inform a full search strategy in PubMed, ERIC, CINAHL, Cochrane Library and Scopus. The key terms and search strategy were developed with assistance of a university librarian and adapted for each included database. Keywords were combined with Boolean operators (Additional File 2). The search was conducted in June 2023 and executed in April 2024.

### Study selection

**Fig. 1:**
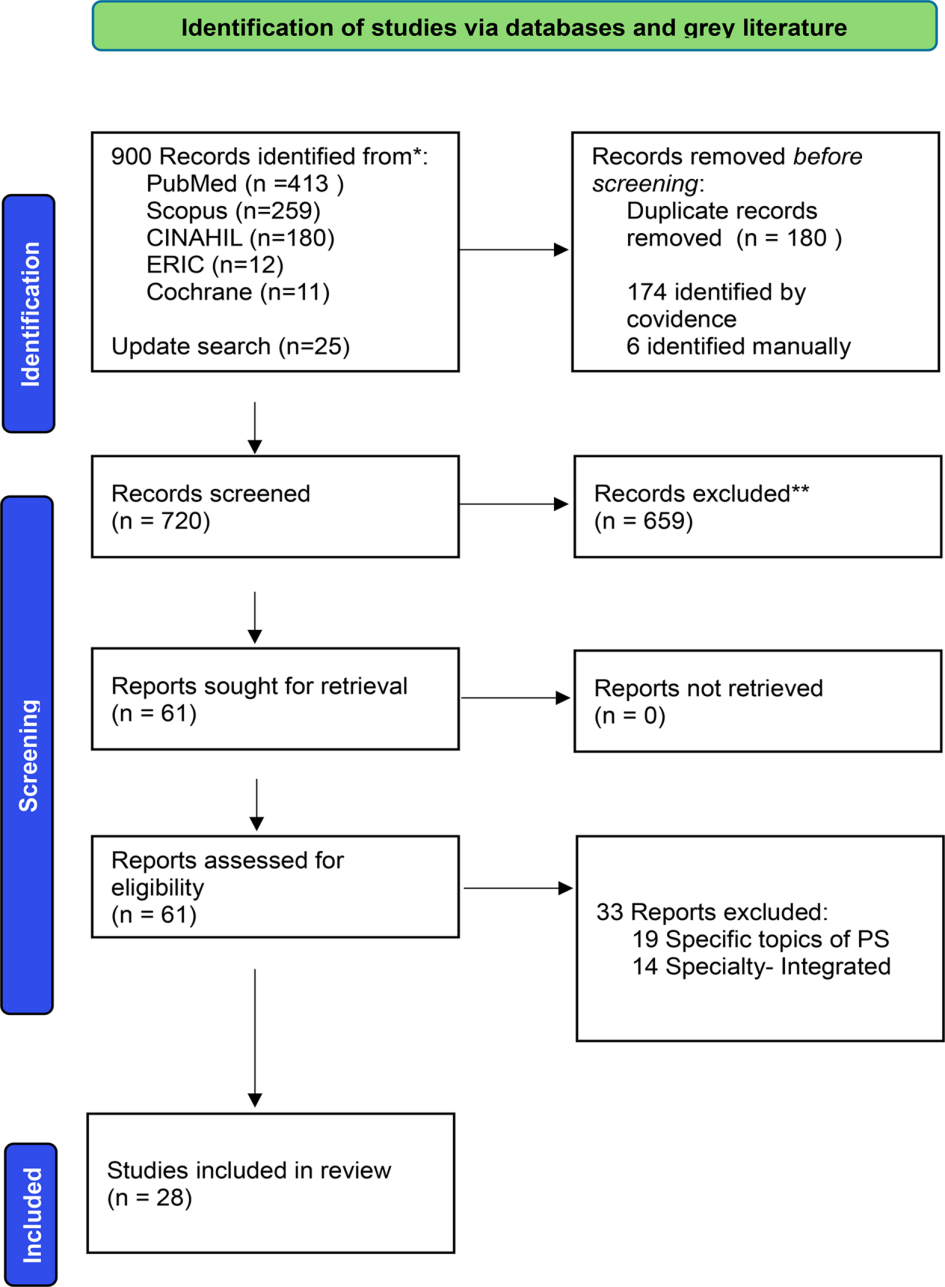
PRISMA-ScR diagram of the review process

900 records were collated and uploaded to *Covidence*, a software for managing reviews. 180 duplicate studies were removed, 720 records were screened at the level of title and abstract, and a further 659 were excluded in a second elimination. 61 papers were retrieved for full analysis, 33 were excluded addressing either specific topics in PSE or speciality integrated. 28 studies were included for the final scoping review. The process and search results are reported using a PRISMA-ScR flow diagram [23] in Figure (1). Titles and abstracts were screened against inclusion criteria by the Principal Investigator (PI) NA and all uncertainties were discussed with second and third reviewers LJ & QA.

### Data extraction

Data extracted from 28 papers by the PI and discussed fully with second researcher LJ. Microsoft Excel spreadsheet was used to capture characteristics (title, author, country, and year); type of evidence; participants; educational intervention; duration of intervention; topics taught; teaching strategies; study design; year level of delivery; and key findings relevant to the review questions (Additional File 3). Extracted data were randomly checked by second and third reviewers (LJ, QA) for accuracy and representation of included studies. Any disagreements that arose between the reviewers were resolved through discussion.

### Data analysis and presentation

Analysis of included articles is reported in two parts. Firstly, descriptive statistics, using simple excel graphics to summarize characteristics of extracted data. Secondly, PI led reflexive thematic analysis which acknowledges the role of researcher(s) in critical reflection [24]. Themes were identified using Braun and Clarke’s six step framework [24] where multiple readings, and highlighting developed data familiarization. Data coding was performed to aggregate repeated insights into codes such as context and location of studies, educational theories, cultural aspects, innovative strategies, staff preparedness. These codes were arranged into initial themes which involved the PI and LJ and refined with QA. The PI drafted the paper with support from LJ and QA.

### Publications characteristics

**Fig. 2:**
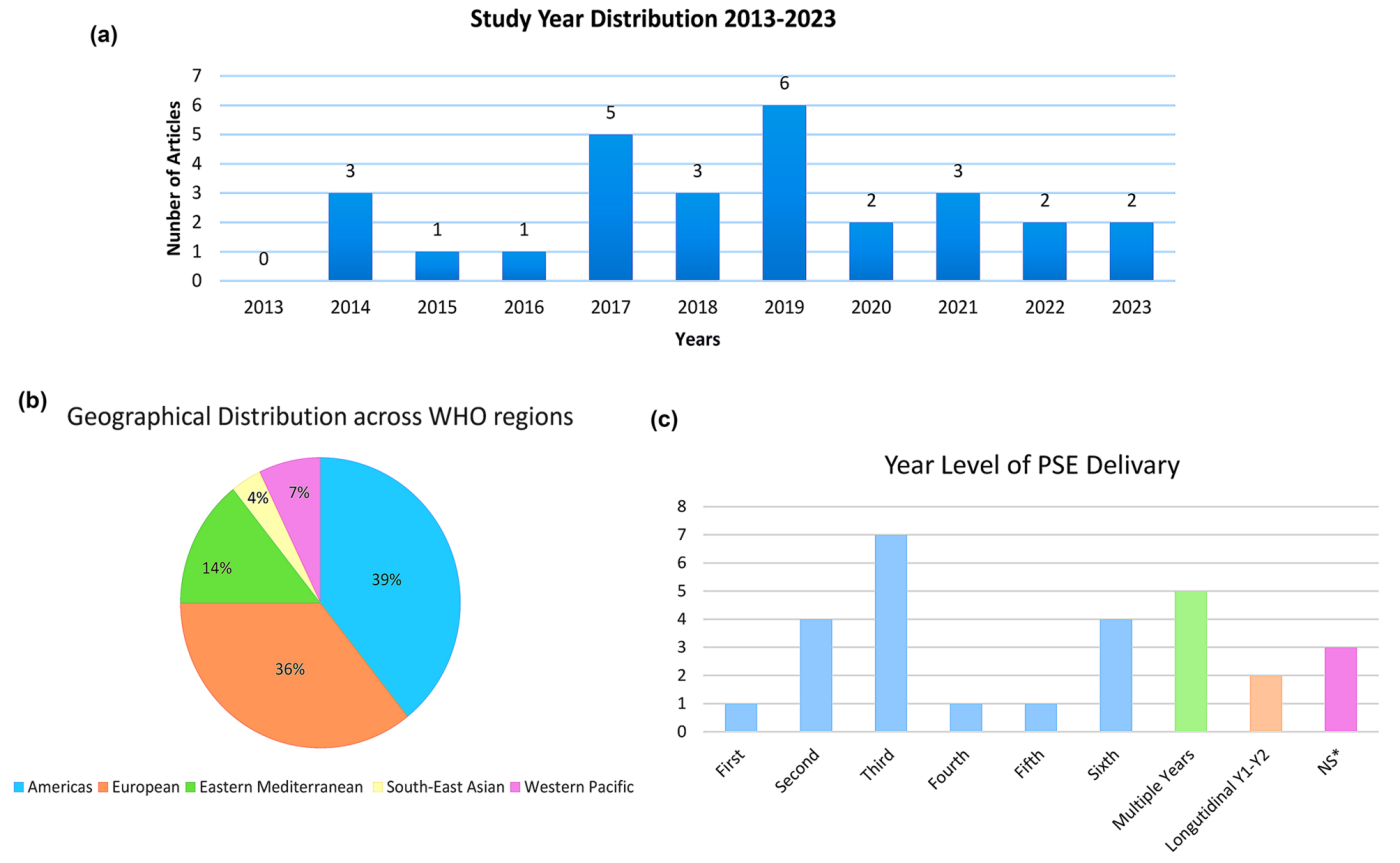
**a**: Study year distribution (2013–2023). **b**: Geographical distribution of publications. **c**: Year level of PSE delivery. *No studies originated from African region. Columns color code: Blue: year of study; Green: multiple years, Orange: longitudinal; Pink: not specified

Selected studies incorporating pedagogic approaches within the last 10 years are graphically represented in (Fig. 2a) to show the time distribution. The majority (*n* = 15) were published in the last 5 years, hinting perhaps at increased attention to creating innovations in teaching approaches.

Distribution of studies across the WHO six regions were: Americas (*n* = 11), European (*n* = 10), Eastern Mediterranean (*n* = 4), Western Pacific (*n* = 2), South-east Asia (*n* = 1), Africa (*n* = 0). For more country specific distribution: USA (*n* = 10), UK (*n* = 4), Germany (*n* = 3) and KSA (*n* = 2), both published in 2021 and 2023, hinting perhaps at recent commitment to PSE in KSA. The remaining publications (*n* = 9) emanated from Australia, Austria, Canada, Egypt, Netherlands, Pakistan, Republic of Korea, Singapore, and Spain. It is noticeable that most papers originated from WEIRD countries with a dearth of publications from non-WEIRD areas (Fig. 2b).

Figure 2c, shows the distribution of when interventions were made by year/level. The most common juncture for delivering educational interventions being during pre-clinical years (*n* = 15) with a peak in 3rd year (*n* = 7). This suggests that medical educators tend to incorporate PSE at the junction between pre and clinical years. None of the selected papers drew on experiences of longitudinal integration throughout the years of the medical program. All were cross sectional or spanning short periods of time [10, 25].

Most studies included were quantitative (61%, 17/28) when compared to qualitative (4%, 1/28). The majority of quantitative studies were pre and post design (*n* = 13), post intervention (*n* = 9), or others (*n* = 6) including comparative study, multi-series, quasi experimental, randomized controlled crossover, prospective non-randomized controlled studies. Of the predominantly quantitative/statistical studies, 75% were empirical, and 25% were pilots. Studies that incorporated qualitative components, such as content or thematic analysis as well as statistics were considered mixed method representing 35% of the studies.

The Kirkpatrick evaluation model, commonly used for program evaluation, was adapted to classify the type of outcome measures including the 4 levels, reaction (L1), learning (L2), behavior (L3), and results (L4) [26]. Reported evaluations utilized one or more measures including satisfaction surveys, self-efficacy questionnaires, feedback forms, interviews (L1), pre and post-knowledge test, reflections (L2), or multi-points behavioral measures (level 3). Most evaluations addressed levels 1–3 with none considering level 4, how did the training affect the organization. This may be an area for future research.

Types of interventions varied from one to multiple sessions (*n* = 7), courses/modules (*n* = 12), workshops (*n* = 3), simulation or discussion-based activities (*n* = 4), seminar (*n* = 1) or longitudinal curriculum (*n* = 2). The duration used for delivering sessions ranged from single 30-miniute activity to multiple sessions/courses spanning between 3 and 17 months. None of the studies included longitudinal integration throughout the UGME program years.

**Fig. 3:**
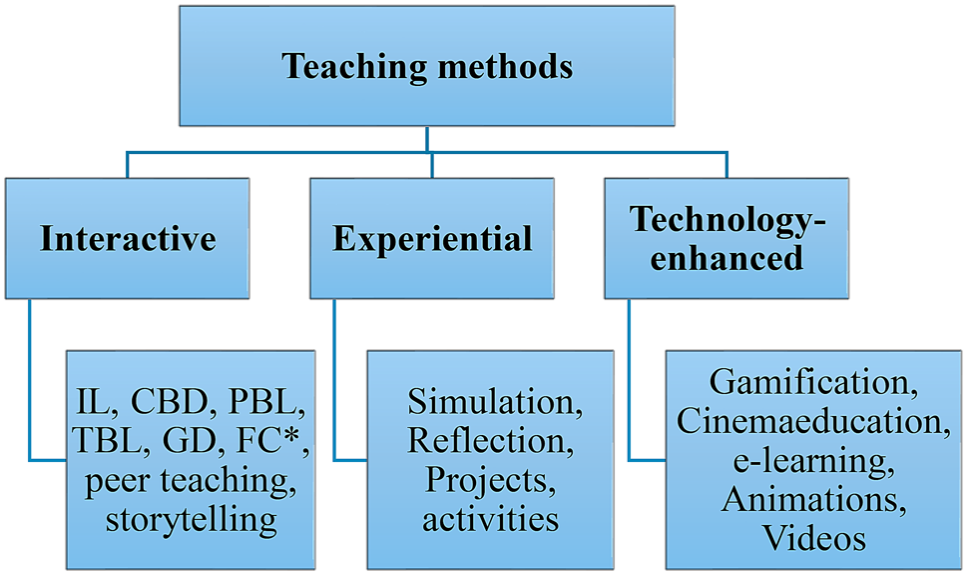
Teaching methods. *IL: Interactive lectures, CBD: Case-based Discussion, PBL: Problem-based learning, TBL: Team-based Learning, GD: Group Discussion, FC: Flipped class

Further analysis examined what teaching strategies were introduced and when. The dataset comprised a total of 17 teaching strategies categorized into interactive (*n* = 8), experiential (*n* = 4), and technology-enhanced strategies (*n* = 5). Interactive teaching strategies included lecture, case-based discussion (CBD), problem-based learning (PBL), team-based learning (TBL), group discussion, flipped classes, peer teaching, and storytelling. Experiential methods included simulation (e.g. role play), hands-on activities, reflection, and group projects (Fig. 3). Statistically, the most used teaching strategy was introductory interactive lectures combined with project-based approaches, case scenarios and group discussions. Most articles (85%) reported combinations of more than one teaching strategy for PSE delivery. Several innovative papers utilized technology-enhanced strategies, simulating working environments whilst engaging students in fun and interactive ways [27]. These include gamification, cinemeducation (using films to facilitate students’ learning [28]), animation, instructional videos, and e-learning suggesting a trend in PSE teaching (Table 3).

**Table 3:** Characteristics of the 28 studies included in the scoping review

Authors, year	Year level	Educational intervention	Duration	Teaching strategies																	Outcome measures	Kirkpatrick level*
				Interactive								Experiential				Technology-enhanced						
				IL	CBD	Peer teaching	PBL	TBL	GD	Flipped Class	Storytelling	Simulation	Reflection	Hands-on activities	Group project	Animation	Cinema education	e-learning	Gamification	videos		
Ahmed et al., 2022	3rd	Course	5 days	✓	✓				✓			✓R		✓							KT*, reflection, CES	2
Arab, 2019	6th	Module	1 semester (15 wks)	✓										✓	✓			✓			KT, satisfaction, self-reported learning	2
Backhouse & Malik, 2019	3rd	1 session	1 h																✓		Feedback forms	1
Baqal et al., 2020	M* (1st -4th)	1 session	1 h			✓					✓					✓					QRE (knowledge/ awareness/perception)	2
Bartlett, & Huerta, 2018	M (1st -4th)	6 sessions	6 months (2 h/session)	✓										✓	✓						Feedback surveys	1
Bawert & Holzinger	NS*	1 session	NS																	✓	QRE (quality) + practical exam	2
Cooper et al., 2019	6th	1 session	NS						✓		✓					✓					QRE (learners’ reactions & perception)	1
Dankbaar et al., 2017	4th	Course	1 wk															✓	✓		students’ satisfaction, knowledge, and self-efficacy awareness	3
Dumenco et al., 2018	1st	Workshop	1 day	✓	✓				✓			✓ R		✓							Satisfaction survey and KT	2
Dumenco et al., 2019	L* (1st & 2nd)	Integrated curriculum	17 months	✓	✓				✓				✓	✓						✓	Knowledge, application- based skills attitudes at 4 points	3
Eltony et al., 2017	6th	Course	3 days		✓		✓		✓				✓	✓							Satisfaction survey and KT	2
Gaupp et al., 2019	3rd	Course	3 months	✓	✓													✓			KT, attitudes questionnaire	3
Gelinas et al., 2021	2nd	Course	6 wks							✓											KT, CES, exam	2
Gheihman et al., 2021	2nd	2 sessions	2 h (1 h/session)		✓								✓								Self-report QRE, reflection, feedback	1
Gonzalez-Caminal, 2023	2nd	Combined activities	2 h									✓					✓				Activity assessment survey	1
Gross et al., 2019	NS	Module	15 min (5/activity)	✓								✓								✓	QRE at 3 points	3
Hayes, 2014	M (1st − 2nd)	Seminar	2 h	✓		✓								✓							Self-efficacy QRE	1
Holderried, 2014	5th	Activity (chart review)	15 h	✓	✓		✓		✓					✓							Hazards identification skills	2
Inayat, 2023	2nd	Course	9 months (2–4 h/wk)	✓					✓						✓						Feedback forms	1
James et al., 2016	3rd	program	1 day		✓			✓				✓						✓			Feedback forms	1
Kow, 2017	3rd	1 session	1 h																✓		APSQ at 2 points	2
Oates et al., 2018	L (1st & 2nd)	8 modules	2 h/each		✓				✓			✓ R								✓	APSQ at 4 points	3
Raty et al., 2017	M (1st -4th)	Course	NS	✓		✓	✓	✓	✓												CES	1
Roh & Kim, 2015	3rd	Course	3 days	✓	✓				✓			✓ R								✓	QRE, Open ended questions	1
Shah et al., 2021	3rd	Activity	50 min	✓					✓												KT, session evaluation	2
shah et al., 2020	M (1st -4th)	workshop	1–2 h each		✓					✓											Questionnaire, Reflection, FB	1
shah et al., 2017	NS	Workshops	NS		✓	✓			✓					✓							Pre-Post survey (knowledge & skills)	2
Thomas et al., 2014	6th	Activity (Sim ward round)	30 min									✓		✓							Pre-Post survey (knowledge, attitude & behavioir)	3

Session content also varied with some focused on providing an overview of general patient safety topics whilst others incorporated cases and scenarios. The main two references frequently used to shape the content of the sessions were the IHI open School (*n* = 11) and/or WHO curriculum guide (for medical schools/multi-professional) (*n* = 7). Other references (*n* = 10) included experts’ consultation, the literature, or were not specified.

### Qualitative analysis

**Fig. 4:**
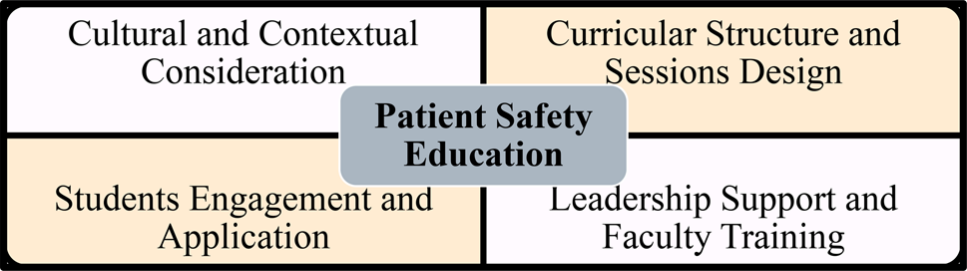
Key themes in PSE discourses

The key themes and patterns identified below were informed by Braun & Clarke’s six step analytic framework [24]. Different PSE discourses were identified by multiple readings and color coding. Four key themes were cultural and contextual considerations; curriculum structure and sessions design; student engagement and application; and leadership support and faculty training (Fig. 4).

#### Cultural and contextual considerations

PSE is highly contextual. Given the diverse nature of healthcare systems globally, the WHO curriculum guide was designed to fit within various cultures and resources availability [13]. This implies that it is the role of educators to consider their local healthcare system context, and align regulations, policies, and guidelines. The WHO curriculum guide points towards the need for modifying clinical cases to fit within the local context of students’ environment [12]. Of the twenty-eight papers reviewed, several authors —albeit from predominantly WEIRD countries — referenced their own experiences, drawing out developments, common mistakes, or sentinel events in their contexts. For example, a German study developed fictional patient charts that highly resemble the ones utilized in their teaching hospital and incorporated the common patient hazards as educational material [29].

Regulations and standards in the UK (GMC) and Germany (GMA) [30] provide guidance for teaching patient safety in their respective contexts. Equivalent national documents highlighting PS competencies are not yet available in some non-WEIRD countries who may have to rely upon examples and evidence from distinctly different contexts and cultures. Few discourses consider local cultures when adding PSE into their educational content [10, 31, 32] or in designing students’ projects [33].

The consensus running through healthcare literature suggests a culture of safety is a foundational aspect of patient care, however, maintaining this culture is a global challenge. Some cultures, or hospitals, lack systems for reporting errors, and even when in place, healthcare providers may tend to overlook them [16]. We found few academic papers that problematized the level of cultural challenge for safe practice for non-WEIRD countries [34]. One exception is a systematic review [35] which has identified that Arab medical practitioners typically believe that there is still a “blame culture” interfering with the reporting of incidents. Low-resourced countries may experience more adverse events than highly resourced western countries due to the lack of information technology advancement, medical knowledge, and financial resources [36]. There is little within the current literature that guides educators, in non-WEIRD countries, on how to bring patient safety to the fore for the next generation of clinicians even when policy makers are promoting a patient safety culture.

#### Curriculum structure/ sessions design

Patient safety is an applied science. Multiple theories and models can inform its design and delivery. At the developmental stage, two of our selected studies used Kern’s six steps of curriculum development [25, 37] which include problem identification, needs assessment, goals and objectives, teaching methods, implementation, evaluation and feedback [38]. One study involved students in the development of the patient safety course [37], another suggested that PSE is best taught through integration across medical curricula [39], however only two selected studies incorporated a longitudinal intervention across first and second year medical students [10, 25].

Experiential Learning Theory (ELT), based on the concept of constructing knowledge from authentic life experiences [40], has been linked to PSE as students have opportunities to participate in various activities mimicking real-life healthcare scenarios. ELT informed the design of three educational interventions. Two German studies incorporated Kolb’s four-mode experiential learning cycle: Concrete Experience, abstract conceptualization, reflective observation, and active experimentation, to design their sessions. The first conducted a session integrating simulation, videos and debriefing [41], while the other designed patient chart review [29]. This cycle allowed students to experience, reflect and apply what they have learnt. Another study in the USA, combined principles of adult learning theory and ELT to co-construct a session on identifying system failure with analysis [42]. Again, these approaches to curricular design have emerged from WEIRD contexts. There is less evidence of adaptation for more diverse contexts.

#### Student engagement and application

There is a wide range of traditional and contemporary teaching methods used in PSE. Several of the reviewed papers considered the importance of creating interactive and engaging learning environments using one or more pedagogic approaches for delivery [43]. For instance, Backhouse & Malik [27] found that gamification, specifically an escape room using a series of cases provided students with new knowledge and skills in enjoyable ways. Students’ feedback identified this escape room method as beneficial for their knowledge on patient safety and general practice. In another study, authors created a pedagogical tool combining simulation and cinemeducation to recreate complex medical professional circumstances [44].

Some studies reported student-led projects as part of educational interventions [33, 45, 46]. Student projects on Quality Improvement (QI) *and* PS were found to be an engaging method as they created hands-on experience to mitigate safety issues noted by students and provide opportunities to think as leaders to find and implement solutions [46].

Narrative pedagogy is evolving as PSE teaching strategy. Two studies reviewed integrated story-based education. In KSA, a study demonstrated that students exhibited improved learning outcomes and higher levels of engagement when exposed to story-based, peer-led PSE sessions featuring scenarios incorporating errors and negative consequences [47]. Another study introduced a unique blend of storytelling and technology, employing animated videos showcasing adverse events encountered by junior doctors [48]. Student feedback of learning about adverse events using animation discussed with near-peers was engaging and effective [48].

Other pedagogical approaches utilized teaching strategies including CBD, where students actively participated in tackling real-life scenarios [42, 49]; SGD [46, 50], flipped classrooms [51], TBL [52, 53] and PBL [29]. Most studies reported enhanced student engagement and understanding in PSE topics. Overall, combining various teaching strategies was prevalent and considered a successful approach for effective delivery of PSE content.

#### Leadership support and faculty training

Universities are often characterized by strong bureaucratic systems, which might impede modifications to existing structures [15]. Limitations and challenges for implementing PSE include resistance to change [15], lack of trained faculty [54], and a misplaced assumption that patient safety must be taught in isolation from other subjects.

According to the WHO, only 14% of nations reported having enough training capacity, indicating a severe global lack of patient safety educators [5]. The literature highlights a shortfall of staff experienced and qualified to teach patient safety [33]. Despite the growth of PS courses in WEIRD countries, only one USA study identified faculty preparedness ahead of teaching PS. This faculty preparation included completion of IHI modules and AAMC teaching for quality (TeQ4) [25].

To address this shortfall in experience and expertise, one study recruited faculty from different medical specialties *and* a lawyer with medicolegal experience to teach their patient safety course [33]. Another intervention was led by formally qualified staff in QI/PS, and delivered to students by residents and attendings from internal medicine and surgery [45]. A study by Shah et al. reported that using peer-to-peer teaching style is not only perceived engaging, but also implies another potential teaching model to overcome the lack of faculty shortage [54]. A study by Raty et al. assigned Residents Teaching Assistants (RTAs) to deliver PSE for undergraduates [53].

## Discussion

### Newcomers to PSE

Undertaking this review, we glimpsed discourses around a growing evidence-base of PSE, noticing how the literature, predominantly originates from WEIRD countries and illuminates the dearth of research from non-WEIRD countries. Our findings suggest that PSE research is strengthening worldwide and more studies may be expected in the future. Despite few contributions from non-WEIRD regions, such as the Arabic speaking diaspora, inclusion of four papers and the PI’s experience of prioritizing and implementing PSE locally, suggest growing commitment to addressing the gap. For instance, KSA, having recognized the gap, is now committed to developing effective teaching and researching of PS [55]. Arguably, pointing a way for non-WEIRD countries to build upon global advances whilst aligning with local cultural and educational practices.

The literature suggests some broad systemic changes are required to support modification to curricula. This is supported by previous studies that found blame culture, workload/inadequate staffing, and poor communication to be key factors hindering positive patient safety culture. These authors suggest supportive leadership, fully vested in implementation of PSE, need effective communication with staff and generation of strength factors. Such factors include supportive organizational attitudes to learning/continuous improvement, good teamwork within units and support from hospital management for patient safety [56]. Another study from KSA identified a correlation between a culture of blame and numbers of medical errors reporting 91% of patient safety errors from 2012 to 2015 were defined as being preventable [57].

It is imperative that managers and leaders engage in strategic planning to safeguard healthcare facilities from potential safety-critical events [58]. Change management might include five key principles - formulating a framework for organizations to guide change process effectively; these include “planning and preparation, communication, stakeholder engagement, training and development, and monitoring and evaluation” [59]. Arguably, change leaders need an evidence-based faculty development strategy as there seems to be a gap in relation to faculty preparation for teaching and researching PS.

### Diverse approaches to PSE

There are many ways to peel an orange. The review shows multiple sources and varieties of pedagogic methods utilised for designing and delivering patient safety curricula. Nie et al. [60], in 2011, suggested further research is needed to identify the best ways to introduce and integrate PS curricula. We acknowledge the many innovative and engaging approaches recently developed, but still, a decade later, we echo their conclusions. Some novel approaches may come from late adopters of the patient safety agenda. For example, story-based and problem-based approaches were utilised in non-WEIRD countries [47, 50]. Could we all benefit from new scholarly contributions to the international discourses that help us understand the different challenges of implementing a culture of patient safety in low- and middle-income countries?

Our review identifies limited evidence of PS driven curriculum change being *explicitly* informed by educational theories. As Bleakley et al. suggest standards and practices in international medical education are more western than truly-global [61]. Use of theory in education has been likened to prescription of drugs. It is important to understand the mechanism of action in both scenarios. Just like knowing how a drug works helps in effective prescribing, understanding how an educational intervention works can lead to choosing optimal approaches for learners in specific contexts [62]. The AMEE guide on experiential learning emphasized how medical educators can bring socio-cultural perspectives to bear on their educational practice [40]. Another scoping review highlighted the potential of using learning theories to inform QI/PS educators, guide pedagogic approaches and curricula modification such as cognitive, sociocultural, transformative and organizational theories [63].

Arguably, there seems to be a gap in the current literature considering *how* to integrate a western evidence base with distinctly different local cultures. One socio-cultural aspect is the difference between individualistic and collectivist societies, as described by Hofstede [64]. Chionis and Karanikas [65] emphasized how the success of safety training is often based on conditions that may vary dependent on sociocultural norms. Late adopters of PSE maybe well-placed to explore and report cultural and contextual factors for consideration moving forward.

There is a consensus in the literature that patient safety is central to effective healthcare delivery and evidence-based PSE curricula guidance and teaching strategies exist globally. One size, however, may not fit all. There is limited evidence relating to implementation of undergraduate PSE in non-WEIRD countries. As more non-WEIRD countries adopt patient safety agendas, we might begin to fill gaps in local, culturally appropriate evidence-based educational practice. Alternative approaches to curricular change or affordable culturally appropriate teaching strategies may be needed, especially in countries with lower incomes, different perspectives on hierarchy, or collectivist cultures. The time may be ripe for not only focusing on promoting education safety cultures in undergraduate curriculum but also for the lived experiences of learners, teachers, or geographical position of undergraduate programs to enhance the evidence base. For instance, issues concerning the safety of conveying medical instructions or prescriptions to illiterate patients can be incorporated into the curriculum where illiteracy prevails.

### Integration of PSE

The World Federation for Medical Education maintains that “*Patient safety is a core attitude and thus needs to be introduced early in medical education and then reinforced throughout postgraduate education and continuing professional development*.*”* [15]. The literature suggests that incorporation of PS teaching should start early in the program [15, 66]. We found limited evidence of why or how this strategy of early introduction of PSE works. Neither did reviewed papers address integration of PSE across all the years of UGME programs. The same gap exists in relation to evaluating benefits of imbedding PSE and its impact on organizations, (Kirkpatrick L4) [26].

Health educators are moving toward patient-focused systems and driving changes towards patient safety cultures within organizations [67]. We, however, found few studies relating to faculty preparedness arguably necessary to promote an attitudinal change and deliver redesigned curricula that integrate PSE. Effective change management requires “buy-in” from educators to enhance their expertise, establish more connections, and involve patients and families [67]. New adopters might build on faculty development and curriculum design strategies emerging from more experienced nations and contributing pedagogic approaches best suited to their own local contexts.

### The value of qualitative research into PSE

Qualitative research “explores and provides deeper insights into real-world problems” [68] perhaps better aligned with the dilemmas of recent adopters. Yet only one qualitatively analysed study was identified [27]. Most of the predominantly quantitative studies did not consider cultural perspectives or how evidence might be adapted for diverse local contexts. This suggests a gap relating to exploration of what might work, and why, in non-WEIRD diverse settings.

### Future research

Mechanisms for integrating PSE aligned with economic and cultural realities seems to be a promising area for further research. For optimal global implementation stakeholder perceptions of the effectiveness of longitudinal or spiral integration of PSE throughout curricula could be highly beneficial. Explicitly combining and problematising evidence-based educational theories with strategies for designing and delivering PSE, might enhance stakeholder understanding of cultural and contextual factors. What influences successful PSE implementation at national and organizational levels? How might local faculties be prepared and developed to implement such changes? More research on evaluating effectiveness of pedagogic interventions against patient safety learning outcomes and competencies would be valuable.

Limitations of this review include valuable papers addressing PSE within postgraduate and interprofessional arenas, were beyond the scope of our study; as were innovative teaching approaches pertaining to specific topics/procedures, such as laryngoscopy in ENT. By limiting our criteria to only English publications, we risked missing non-English papers, however, evidence-base from non-WEIRD countries are predominantly in English. Only the PI screened the items against title and abstract. To mitigate this limitation, all uncertainties of inclusions were discussed with the other authors who also randomly sampled the PI’s analysis finding only minor discrepancies. This scoping review was conducted as one stage of a PhD specific to studying how to effectively implement integration of basic PS principles into undergraduate medical curricula in middle and far eastern countries. It has already informed the empirical design, aligned with the aims of the JBI scoping review protocol, having been used to justify and shape a qualitative empirical study focusing on UG medical curricula in a Saudi (non-WEIRD) and Dundee (WEIRD) countries.

## Conclusions

This scoping review highlights gaps in the literature on preparing safe practitioners and mitigating errors, particularly in non-WEIRD countries. It has identified the need for faculty development and emphasizes the importance of considering cultural perspectives and adapting evidence for diverse local contexts in PSE. There is limited evidence of the efficacy of how and when PS is delivered across UG programs globally, longitudinal studies, or how these curriculum changes impact medical educational institutions.

The literature illuminates how early adopters, mostly WEIRD countries, have led and supported the integration of PS for decades and developed an evidence-base useful for late adopters. The review however does challenge assumptions that this evidence-base will automatically fit all countries and contexts and suggests more educational theory of *how and when* PSE is delivered could enrich international discourses. As the number of studies from non-WEIRD countries grows, our understanding of how to promote, create and evaluate a culture of PSE across diverse countries can expand. The range of evidence-based pedagogic interventions available may also increase.

## Electronic supplementary material

Below is the link to the electronic supplementary material.

Supplementary Material 1: PRISMA-ScR checklist

Supplementary Material 2: Search Strategy for various databases

Supplementary Material 3: Data extraction of the findings

## Data Availability

No datasets were generated or analysed during the current study.

## Abbreviations

PS: Patient Safety
QI: Quality Improvement
UG: Undergraduate
UGME: Undergraduate Medical Education
PSE: Patient Safety Education
WEIRD: Western, Educated, Industrialized, Rich and Democratic
IHI: Institute of Healthcare Improvement
WHO: World Health Organization
ELT: Experiential Learning Theory
