# Supplementary material for: Patient safety education in undergraduate medical education through a global lens: a scoping review
Source: BMC Med Educ. 2025 Apr 16;25:544. doi: 10.1186/s12909-025-07159-x (PMC12001576; doi:10.1186/s12909-025-07159-x)
Supplement: Supplementary file 3 — Supplementary Material 3: Data extraction of the findings [file 12909_2025_7159_MOESM3_ESM.docx]

**Search strategies version 2. 20^th^ March 2023**

The following searches use a mix of controlled index terms specific to the particular database and keywords relevant to the topic. These have been combined with Boolean operators.

**PubMed via PubMed.gov**

|  | Patient safety[MeSH Terms] |
| --- | --- |
|  | "Patient safety"[Title/Abstract] |
|  | 1 or 2 |
|  | Students, medical[MeSH Terms] |
|  | "medical students"[Title/Abstract] |
|  | "medical undergraduates"[Title/Abstract] |
|  | 4 or 5 or 6 |
|  | Integrated[Title/Abstract] |
|  | Curriculum[Title/Abstract] |
|  | "Medical education"[Title/Abstract] |
|  | Teaching[Title/Abstract] |
|  | Education, Medical, Undergraduate[MeSH Terms] |
|  | 8 or 9 or 10 or 11 or 12 |
|  | 3 and 7 and 13 |

((Patient safety[MeSH Terms]) OR ("Patient safety"[Title/Abstract])) AND (((Students, medical[MeSH Terms]) OR ("medical students"[Title/Abstract])) OR ("medical undergraduates"[Title/Abstract]))) AND ((Curriculum[Title/Abstract] OR "Medical education"[Title/Abstract] OR Teaching[Title/Abstract] OR Integrated[Title/Abstract]) OR (Education, Medical, Undergraduate[MeSH Terms]))

**ERIC via Ebsco**

|  | Patient safety |
| --- | --- |
|  | DE "Medical Students" |
|  | Medical students |
|  | Medical undergraduates |
|  | 2 or 3 or 4 |
|  | DE "Medical Education" |
|  | Medical Education |
|  | Medical curriculum |
|  | Medical teaching |
|  | Integrated |
|  | 6 or 7 or 8 or 9 or 10 |
|  | 1 and 5 and 11 |

**Scopus via Elsevier**

(TITLE("patient safety") AND TITLE-ABS-KEY (medical AND (student* OR undergraduate*)) AND TITLE-ABS-KEY (medical AND (curriculum OR teaching OR education OR integrated)))

**Cochrane Library via Wiley**

#1 MeSH descriptor: [Patient Safety]

#2 MeSH descriptor: [Students, Medical]

#3 "medical students" OR "medical undergraduates"

#4 MeSH descriptor: [Education, Medical, Undergraduate]

#5 medical (curriculum OR teaching OR education OR integrated)

#6 #2 OR #3

#7 #4 OR #5

#8 #1 AND #6 AND #7

**CINHAL Plus via Ebsco**

|  | (MH "Patient Safety") |
| --- | --- |
|  | Patient safety |
|  | 1 or 2 |
|  | (MH "Students, Medical") |
|  | medical students |
|  | medical undergraduates |
|  | 4 or 5 or 6 |
|  | (MH "Education, Medical") |
|  | Medical education |
|  | Medical curriculum |
|  | Medical teaching |
|  | Integrated |
|  | 8 or 9 or 10 or 11 or 12 |
|  | 3 and 7 and 12 and 13 |
